# TRIM21-mediated Sohlh2 ubiquitination suppresses M2 macrophage polarization and progression of triple-negative breast cancer

**DOI:** 10.1038/s41419-023-06383-x

**Published:** 2023-12-20

**Authors:** Ruihong Zhang, Ying Shen, Qi Zhang, Xiaoning Feng, Xuyue Liu, Xiaoning Huo, Jinhao Sun, Jing Hao

**Affiliations:** 1 Key Laboratory of The Ministry of Education for Experimental Teratology, Department of Histology and Embryology, School of Basic Medical Sciences, Cheeloo College of Medicine, Shandong University, 44 Wenhua Xi Road, Jinan, Shandong 250012 China; 2 Department of Human Anatomy and Neurobiology, School of Basic Medical Sciences, Cheeloo College of Medicine, Shandong University, 44 Wenhua Xi Road, Jinan, Shandong China

**Keywords:** Transcriptional regulatory elements, Cell growth

## Abstract

Lung metastasis is the major cause of death in patients with triple-negative breast cancer (TNBC). Tumor-associated macrophages (TAMs) represent the M2-like phenotype with potent immunosuppressive activity, and play a pro-tumor role in TNBC lung metastasis. Sohlh2 belongs to the basic helix-loop-helix transcription factor family. However, its role in macrophages polarization remains unknown, especially in TNBC progression. Here we demonstrated that Sohlh2 overexpression promoted M2 macrophage polarization. Moreover, high expression of Sohlh2 in M2-like macrophage enhanced TNBC cell growth, migration and lung metastasis in vivo and in vitro. Mechanistically, we revealed that Sohlh2 functioned through up-regulating LXRα, ABCA1, ABCG1 expression and disturbing the lipid homeostasis on the membrane of macrophages. Sohlh2 could directly bind to the promoter of LXRα and promote its transcription activity. E3 ubiquitin ligase TRIM21 promoted Sohlh2 ubiquitination and degradation, and suppressed M2 macrophage polarization and TNBC progression. Collectively, our findings suggested that Sohlh2 in macrophage could be a novel therapeutic target for TNBC metastatic treatment.

## Introduction

Triple-negative breast cancer (TNBC), also known as estrogen receptor-negative (ER-), progesterone receptor-negative (PR-), and human epidermal growth factor receptor 2-negative (HER2-) breast cancer, accounts for 15~20% of newly diagnosed breast cancers [1, 2]. Compared with other breast cancer subtypes, TNBC is characterized by its high histological grade, and early distant metastasis. Due to its aggressive clinical features and the lack of effective therapeutic targets, TNBC has a poor prognosis. Although some new methods have been recently tried to treat TNBC, they are not very effective[3, 4]. Thus, addressing novel molecular mechanism driving TNBC progression and metastasis is urgently needed [5, 6].

Tumor-associated macrophages (TAMs) can be polarized into two different states, either antitumor M1 macrophages that express inflammatory factor, such as TNFα, IL-6, IL-8, and chemokines, such as CXCL10, or protumor M2 macrophages that synthesize and release anti-inflammatory cytokines, immunosuppressive factors, and various cytokines such as IL-10, TGFβ, VEGFA. These factors promote tumor angiogenesis and extracellular matrix degradation and remodeling, which in turn promote tumor cell migration, invasion and distant metastasis [7–10]. Recent studies implicate that macrophage functions are influenced by cellular metabolism, including lipid metabolism[11, 12]. Macrophage cholesterol metabolism is closely associated with TAM polarization [13, 14]. The liver X receptor alpha (LXRα), a key regulator of cholesterol, fatty acid, and glucose metabolism, belongs to the nuclear receptor superfamily and is highly expressed in the liver, intestine, adipose tissue, and macrophages [15–17]. Upon ligand activation, LXRα forms a heterodimer with the vitamin X receptor (RXR) and regulates the expression of target genes containing LXR response elements [11]. LXR regulates reverse cholesterol transport by controlling the expression of cholesterol transporters and apolipoproteins, such as ATP binding cassette subfamily A member 1 (ABCA1), ATP binding cassette subfamily G member 1 (ABCG1), apoE, and apoC [18–20]. Macrophages highly express ABCA1 and ABCG1 to promote intracellular cholesterol efflux and maintain lipid homeostasis at the cell membrane [21, 22]. The deficiency of ABC transporters could hinder membrane cholesterol efflux in macrophages and inhibit tumor progression and metastasis [23, 24].

Sohlh2 belongs to the superfamily of basic helix-loop-helix (bhlh) transcription factors [25]. Preliminary results showed that Sohlh2 was expressed in macrophages, and affected the polarization of macrophages. The underlying mechanism of Sohlh2 in macrophage polarization requires further exploration.

Here in this study, we utilized co-culture of macrophages and TNBC cells, macrophage-specific Sohlh2 conditional knock-in mice to identify the important role of Sohlh2 in macrophages polarization and progression of TNBC cells in vivo and in vitro. Sohlh2 promoted the M2-type polarization of macrophages by regulating macrophage cholesterol efflux via the activation of LXRα/ABCA1, ABCG1 signaling pathway, and then induced the metastasis of TNBC cells. TRIM21 mediated the ubiquitination modification and degradation of Sohlh2 protein, and suppressed macrophage M2 polarization and TNBC progression. This may be a novel mechanism by which TRIM21 reverses the M2 type polarization of macrophages caused by Sohlh2/LXRα signaling pathway and subsequently prevents TNBC progression.

## Materials and Methods

### Cell lines and cell culture

Human and mouse triple-negative breast cancer cell lines MDA-MB-231 and 4T1.2, human and mouse monocyte cell lines THP-1 and RAW264.7, and HEK 293 T were purchased from the Cell Banks of Type Culture Collection of Chinese Academy of Sciences (Shanghai, China) and were cultured in PRMI 1640 supplemented with 10% FBS and 100U/ml Penicillin and 100 µg/ml Streptomycin in a humidified incubator of 5% CO_2_ at 37 °C. THP-1 and RAW264.7 cells were differentiated to a macrophage-like state by incubating with 20 nM PMA (APExbio) overnight, then M0 macrophages were polarized into M1 macrophages using LPS (20 ng/ml) (Sigma, USA) plus IFNγ (20 ng/mL) (Peprotech, USA), into M2 macrophages using IL-4 (20 ng/ml) (Peprotech, USA) for 48 h. The cell lines were authenticated by short tandem repeat (STR) profiling and tested free of mycoplasma.

### RNA interference and plasmid transfection

THP-1 and RAW264.7 cells were transfected with Sohlh2 lentiviral GV358 or shRNAs (Genechem, China). Stable-expressing cells were established by puromycin (25 μg/ml, Solarbio) selection. Cells were harvested after puromycin selection for transfection efficiency by qPCR and immunoblotting.

For knockdown of TRIM21 and LXRα, siRNAs against TRIM21 (Genechem, China) or LXRα (siRNA), were transfected into the THP-1 cells using lipofectamine 3000 according to the manufacture’s protocol. The target sequences selected are shown (Table S1).

### Animal studies

All animal studies were conducted in compliance with the Ethics Committee of School of Basic Medical Science of Shandong University. The *Sohlh2*^fl/fl^ mice were constructed by Cyagen using the CRISPR-Cas9 gene-editing system. Lyz2-Cre mice were obtained from Professor Chengjiang Gao Laboratory. *Sohlh2*^fl/fl^ mice were crossed with Lyz2-Cre mice to generate *Sohlh2*^fl/fl^ Lyz2-Cre mice.

To obtain mouse PMs (Peritoneal macrophages), the mice were injected with starch by intraperitoneal injection 3 days in advance, and peritoneal exudate cells were obtained 3 days later. After 2 h, un-adherent cells were removed, adherent monolayer cells were used as PMs.

To obtain BMDMs (bone-marrow-derived macrophages), BMDMs were isolated from the tibia and femur, and cultured with DMEM containing 20% FBS, glutamine and 30% L929 supernatant for 4 days. The cells were further cultured for another 3 days before the harvest of the adherent BMDMs.

4T1.2 cells (5 × 10^6^) were implanted subcutaneously injected into the female mice aged 6 weeks to establish the TNBC lung metastasis model. All mice (5 mice per group) were randomly divided into the control and experimental groups. The phenotype was analyzed by a blind investigator. All mice were imaged and sacrificed for analyzing lung metastasis in vivo.

### Patient specimens

40 cases of primary human TNBC specimens were purchased from Shanghai Superbiotek Pharmaceutical Technology Inc. (Shanghai, China). The histopathological diagnose were determined by expert pathologists, and the study complied with the Ethics Committee of Shandong University.

### Transwell coculture

For coculture with macrophages, 1 × 10^6^ MDA-MB-231 or 4T1.2 cells were seeded in 1 ml RPMI 1640 containing 10% FBS into the upper Transwell chamber (Millipore) and allowed to adhere overnight. The next day, 1 × 10^6^ THP-1 or RAW264.7 cells in 1 ml RPMI 1640 containing 10% FBS were seeded into the lower well and the coculture was incubated for 24 h. Cells were collected for further experiments.

### RNA extraction and RT-qPCR

Total RNA was extracted using TRIZOL reagent (Invitrogen) according to the manufacturer’s instructions and was reverse transcribed using reverse transcriptase (Thermo Fisher Scientific). A LightCycler and SYBR RT-PCR kit (ComWin Biotech) were used for real-time quantitative RT-PCR (qRT-PCR) analysis. The PCR primers are shown in Table S2. Data were normalized to GAPDH expression in each sample.

### Western blot

Cells were lysed in RIPA buffer (Solarbio) containing protease inhibitor cocktails (Sigma) and phosphatase inhibitors. The proteins were separated by SDS-polyacrylamide gel, transferred onto polyvinylidene fluoride membranes (Bio-Rad) that were blocked with 5% dry milk for 2 h, then incubated with primary antibodies overnight at 4 °C, followed by peroxidase-conjugated secondary antibodies (1:5000 dilution) for 1 h at RT. The immune complex was visualized by an enhanced chemiluminescence kit (Millipore). All antibodies used in this study are provided in Table S3.

### Cell migration and invasion assays

For cell migration and invasion assays, 4 × 10^4^ MDA-MB-231 or 4T1.2 cells resuspended in serum-free medium were plated into Transwells (Corning). For cell invasion assays, 5 × 10^4^ cells were resuspended in serum-free medium and seeded into Transwells with Matrigel (Corning). Medium (500 μl) containing 15% FBS was added to the lower chamber. After incubation for 6 to 8 h, the cells in the lower chamber were fixed with methanol and stained with 0.2% crystal violet.

### Cytokine ELISA

The concentration of IL-10, TGFβ and TNFα in the CM or sera of macrophages was measured with the human or Mouse IL-10, TGFβ and TNFα ELISA Kit (ExCell Bio). Coculture CM or mice sera was added to 96-well ELISA plates for 2 h according to the manufacturer’s instructions. All experiments were done in triplicates, and performed three separate times.

### ChIP assay

For the ChIP assay, 1 × 10^7^ THP-1 cells were prepared for ChIP assay following the manufacturer’s instructions (Cell Signaling Technology). Briefly, the chromatin fragments solutions were incubated with 10 µl anti-Sohlh2 antibody or the rabbit IgG and Protein G magnetic beads at 4 °C overnight with rotation state. The precipitated DNAs were eluted, separated by magnetic separator, purified using spin columns, and finally analyzed by qPCR. The specific primer pairs for human LXRα promoter were shown in Table S2.

### Luciferase reporter assay

For the luciferase reporter assay, Sohlh2-overexpression and Sohlh2-knockdown THP-1 cells were seeded into 24-well plates. Cells were transfected with 10 ng pGL3-firefly luciferase plasmids together with 10 ng pRL-Renilla luciferase plasmid and 200 ng LXRα luciferase reporter using Lipofectamine 3000 (Invitrogen). The activities of luciferase were measured by the Luciferase Reporter Assay system (Promega) according to the manufacturer’s instructions. Firefly luciferase activities were normalized to those of Renilla luciferase activities in each sample.

### Filipin staining

Filipin fluorescent staining kit (sigma) was performed to detect free cholesterol according to the manufacturer’s instructions. The cells were covered with a coverslip immediately after 1 h staining and then observed under a fluorescence microscope (Olympus). The cholesterol deposits exhibited blue fluorescence after staining.

### Multiplexed immunofluorescent staining

Multiplexed immunofluorescence staining of tissue microarray (TMA) was performed using Four-color multiplex fluorescent immunohistochemistry kit (absin) according to the manufacturer’s instructions. The paraffin-embedded slices were dewaxed. Slices and cells were permeabilized with 0.1% Triton X-100 and blocked with 5% BSA. Slides were incubated with the primary antibody (Sohlh2, iNOS, CD163 or LXRα) for 1 h, and subsequently incubated with HRP-conjugated secondary antibody for 10 min after removed primary antibody and washed in TBST buffer. Then, slides were incubated with signal amplifier buffer for 10 min at RT. The above procedures were repeated for other antibodies, and antibodies were removed by microwave treatment (1 min 100% power) before another round of staining was performed, then sealed with DAPI. Details of antibodies are described in Table S3.

### Co-immunoprecipitation (Co-IP)

THP-1 or HEK293T cells were collected 24 h after transfection and lysed in lysis buffer supplemented with a protease inhibitor cocktail (Sigma), and a phosphatase inhibitor cocktail (Sigma). Supernatants were collected and incubated with anti-Flag antibody and normal mouse IgG (Sigma) as a negative IP control for 2 h. The mixtures were incubated with protein A/G beads (MCE) overnight at 4 °C. Then, beads were washed 3 times with lysis buffer and eluted by boiling with 2× SDS loading buffer.

### Ubiquitination assay

To assess the ubiquitination of Sohlh2 in THP-1 cells, the cells were transfected with Flag-Sohlh2, HA-Ub, TRIM21 or shTRIM21. Whole-cell extracts were Immunoprecipitated with anti-Flag and analyzed by immunoblotting with anti-HA antibody.

### Mass spectrometry

After staining protein in SDS-PAGE gels with coomassie blue, gel lanes were sliced into different bands. The specific analysis process was performed as the manufacturer’s instructions, and the binding proteins were measured by mass spectrometry (Maxis II, Advanced Medical Research Institute, Shandong University).

### Statistical analysis

All values were expressed as mean ± SEM, and the number of samples (*n*) was indicated. The statistical significance of differences between control and experimental groups was determined by Student *t* test with *P* < 0.05 considered significant (*P < 0.05; **P < 0.01; ***P < 0.001). All statistical analyses were performed using GraphPad Prism v6.0d. More information of the materials and methods is in the Supplementary Materials.

## Results

### Sohlh2 is associated with M2 macrophage polarization

**Fig. 1:**
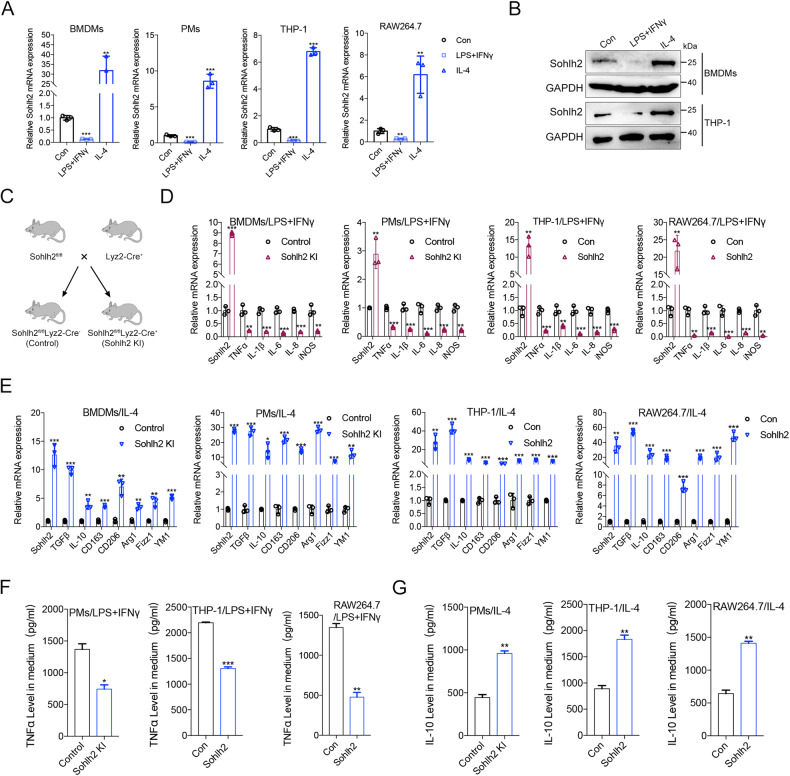
Sohlh2 is associated with M2 macrophage polarization. **A**, **B** qPCR and Western blot assays showed the expression of Sohlh2 in different phenotype polarization of macrophages. **C** Schematic diagram depicting the generation of macrophage-specific Sohlh2 knock-in mice. **D**, **E** The mRNA expression levels of M1 or M2 macrophage-related genes were examined by qPCR. **F**, **G** The secretion of TNFα and IL-10 was measured by Elisa analysis. The values indicate the mean ± SD of three independent experiments. *P < 0.05, **P < 0.01, ***P < 0.001.

To identify the expression of Sohlh2 in different macrophage phenotype polarization, we carried out qPCR and Western blot analysis in macrophages, the expression of Sohlh2 was significantly downregulated in M1 macrophage, while the expression of Sohlh2 was significantly upregulated in M2 macrophage (Fig. 1A, B). To determine the effects of Sohlh2 on macrophage polarization, the expression levels of M1 or M2 macrophage-associated genes were evaluated in the Sohlh2 overexpression and knockdown sable THP-1 and RAW264.7 cell lines or BMDMs and PMs from Sohlh2^fl/fl^ Lyz2-Cre (Sohlh2 KI) mice.

The mRNA expression levels of M1 macrophage-associated genes, including TNFα, IL-1β, IL-6, IL-8, and iNOS were significantly downregulated in Sohlh2 overexpression macrophage (Fig. 1C, D). The mRNA expression levels of M2 macrophage-associated genes, including IL-10, TGFβ, CD163, CD206, Arg1, Fizz1, YM1 were significantly upregulated in Sohlh2 overexpression macrophage (Fig. 1E). Further, We found the secretion of TNFα was significantly reduced in Sohlh2 overexpression macrophage treated with LPS plus IFNγ (Fig. 1F). However, the secretion of IL-10 was increased in Sohlh2 overexpression macrophage treated with IL-4 (Fig. 1G). As shown in Fig. S1, Sohlh2 knockdown in THP-1 and RAW264.7 induced M1 macrophage-associated gene expression and TNFα secretion, whereas reduced M2 macrophage-associated gene expression and IL-10 secretion. Taken together, Sohlh2 polarized macrophage to M2 phenotype.

### Sohlh2 in M2 macrophage promotes the oncogenic properties of TNBC cells in vitro

**Fig. 2:**
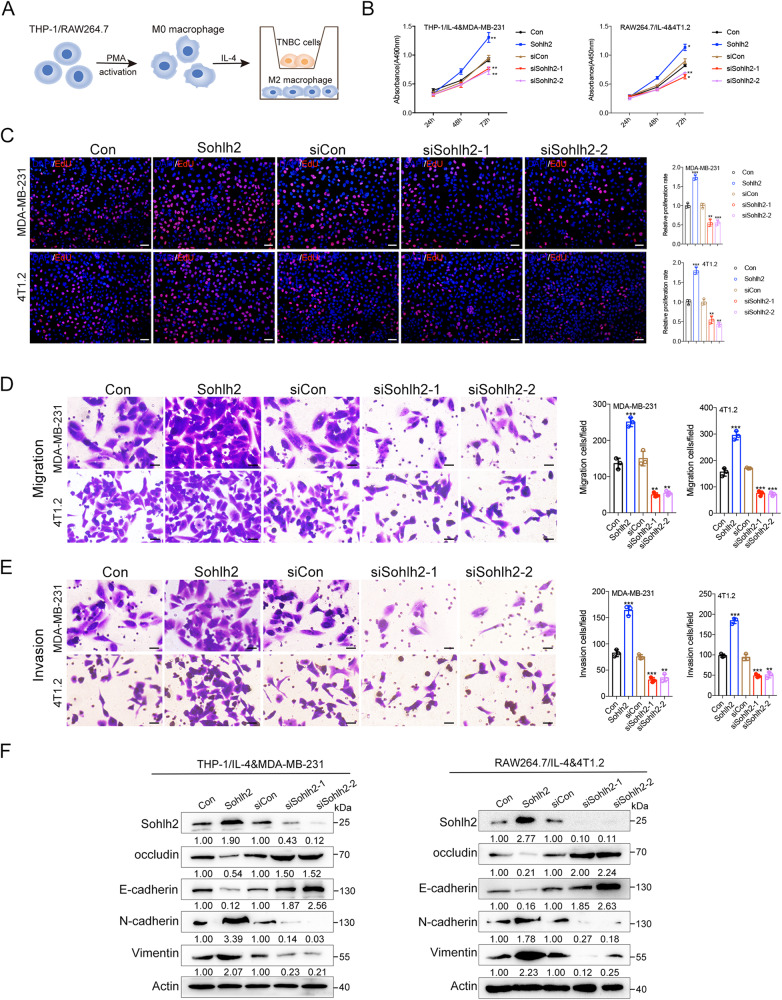
Sohlh2 in M2 macrophage promotes the oncogenic properties of TNBC cells in vitro. **A** Scheme of the workflow illustrating that M2 macrophage co-cultured with TNBC cells in Transwell chambers. The MDA-MB-231 and 4T1.2 cell growth rates were evaluated with CCK8 (**B**) and EdU (**C**) assays. Migration (**D**) and Invasion ability (**E**) were performed in MDA-MB-231 or 4T1.2 cells co-cultured with M2 macrophages by Transwell assays. **F** EMT-related markers in MDA-MB-231 or 4T1.2 cells co-cultured with M2 macrophages were detected by western blot analysis. Scale bar indicates 50 μm. The values indicate the mean ± SD of three independent experiments. *P < 0.05, **P < 0.01, ***P < 0.001.

To evaluate whether macrophage polarization is responsible for promoting the proliferation, migration and invasion of TNBC cells, we co-cultured macrophages with MDA-MB-231 or 4T1.2 cells using Transwell chambers (Fig. 2A). The CCK8 and EdU assays showed that Sohlh2 overexpression in M2 macrophage enhanced MDA-MB-231 and 4T1.2 cell growth capabilities, while silencing of Sohlh2 inhibited the proliferation of TNBC cells (Fig. 2B, C). Moreover, Sohlh2 overexpression in M2 macrophage caused a notable increase in migrated and invaded MDA-MB-231 or 4T1.2 cells, as expected, compared with the siCon group, the number of migrated and invaded MDA-MB-231 or 4T1.2 cells was significantly decreased when silencing Sohlh2 in M2 macrophage (Fig. 2D, E). We also found Sohlh2 overexpression in M2 macrophage increased the expression of mesenchymal markers, while inhibited the expression of epithelial markers in TNBC cells (Fig. 2F). However, knockdown Sohlh2 had the opposite results (Fig. 2F).These results indicate that Sohlh2 in M2 macrophage promotes the oncogenic properties of TNBC cells.

### Sohlh2 in M2 macrophage accelerates TNBC progression in vivo

**Fig. 3:**
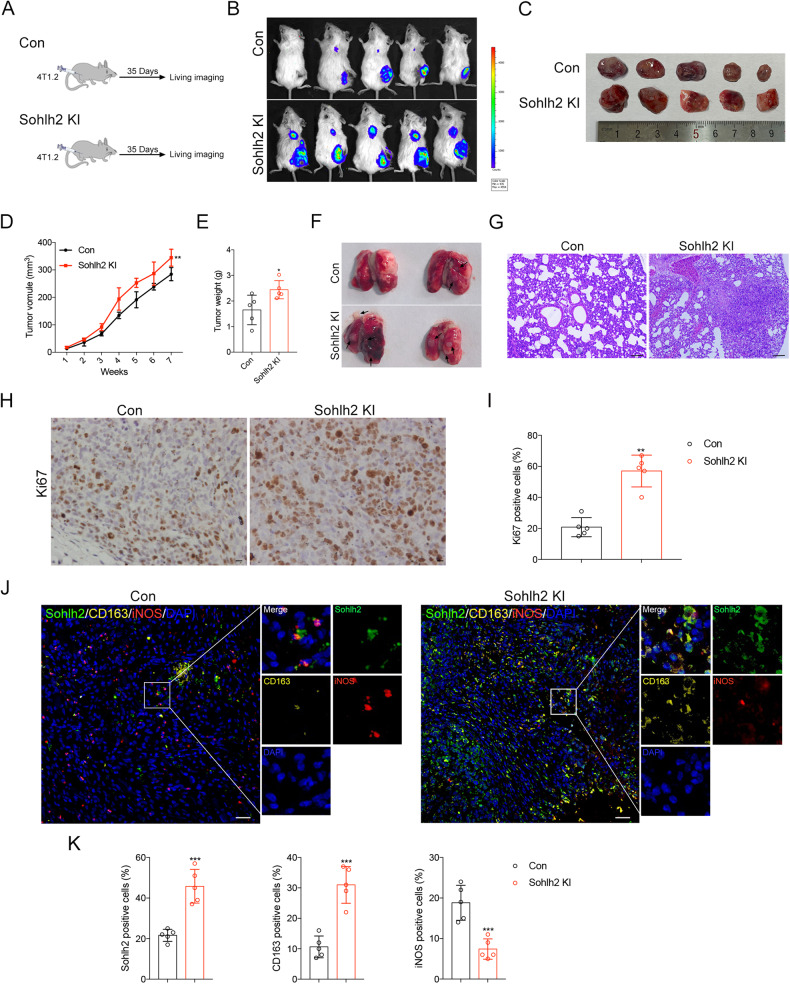
Sohlh2 in M2 macrophage accelerates TNBC progression in vivo. **A** Schematic diagram of TNBC lung metastasis following subcutaneous injection with Sohlh2 stably expressing or the control 4T1.2 cells. **B**, **C** Lung metastasis and the subcutaneous tumors were shown by live imaging in mice. After subcutaneous injection of 4T1.2 cells containing a stable overexpression of Sohlh2 or the control, tumor growth curves are shown (**D**). The tumor weights (**E**) were measured when the mice were sacrificed and the tumors were excised. **F**, **G** Lung metastatic lobules were shown and detected by HE. Scale bar indicates 100 μm. **H**, **I** The growth capacity of subcutaneous tumor cells was demonstrated by Ki67 staining. Scale bar indicates 20 μm. **J**, **K** Multiplexed Immunofluorescence staining was performed to detect the expression of Sohlh2, CD163 and iNOS in subcutaneous tumors. Scale bar indicates 50 μm. The values indicate the mean ± SD of three independent experiments. *P < 0.05, **P < 0.01, ***P < 0.001.

To further assess the effects of Sohlh2 in M2 macrophage on tumor growth and metastasis, 4T1.2 cells were inoculated subcutaneously into female Sohlh2 KI and Con mice (Fig. 3A). The tumor growth of Sohlh2 KI xenografts was markedly promoted as demonstrated by the increased volume (Fig. 3B–D) and weight (Fig. 3E) compared with the Con group. We observed a strong increase of lung metastasis in Sohlh2 KI mice (Fig. 3F, G). IHC assay showed overexpression of Sohlh2 led to significant increase of the Ki67 positive cells in xenografts (Fig. 3H, I). Interestingly, we found that macrophage-specific Sohlh2 KI led to more CD163 positive TAMs in xenografts and less expression of iNOS in macrophages (Fig. 3J, K). Taking together, these findings suggest that Sohlh2 in M2 macrophage accelerates TNBC progression in vivo.

### Sohlh2 activates LXRα/ABCA1, ABCG1 signaling pathway and increases lipid efflux in macrophage

**Fig. 4:**
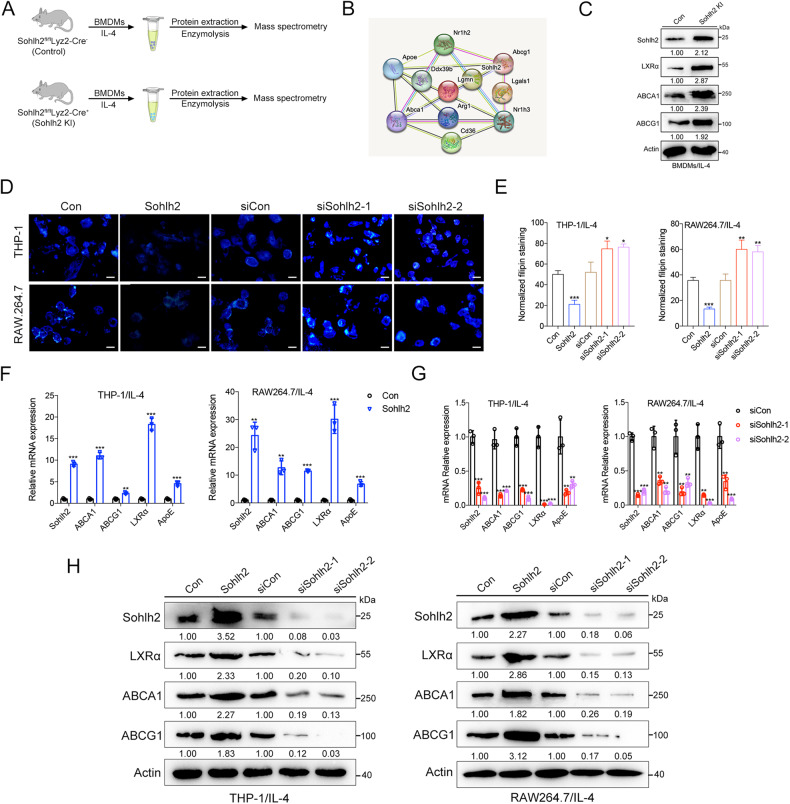
Sohlh2 activates LXRα/ABCA1, ABCG1 signaling pathway and increases lipid efflux in macrophage. **A** Differential proteins were detected by mass spectrometry in BMDMs from the Control or Sohlh2 KI mice. **B** Diagram of the interactions of the top 11 upregulated genes in BMDMs from Sohlh2 KI mice. **C** Western blot analysis of LXRα, ABCA1 and ABCG1 in BMDMs. **D**, **E** Filipin staining was performed to analyze the cholesterol sediment. qPCR (**F**, **G**) and Western blot (**H**) assays showed the expression of LXRα, ABCA1 and ABCG1 in M2 macrophages. Scale bar indicates 20 μm. The values indicate the mean ± SD of three independent experiments. *P < 0.05, **P < 0.01, ***P < 0.001.

Macrophage membrane cholesterol metabolism plays an important role in the progression of tumor. To exploit the effect of Sohlh2 on cholesterol metabolism in M2 macrophage, mass spectrometry was performed to investigate the differential gene expression in BMDMs from Sohlh2 KI and Con mice (Fig. 4A). Top 11 upregulated genes and their interactions in Sohlh2 KI BMDMs were shown in Fig. 4B, in which LXRα was the highest upregulated gene(Fig. S2A). qPCR and Western blot results confirmed that Sohlh2 expression in BMDMs was higher in Sohlh2 KI mice compared to Con mice (Figs. S2B, 4C). The results of filipin staining showed overexpression of Sohlh2 decreased cholesterol deposits, while Sohlh2-knockdown increased the cholesterol deposits (Fig. 4D, E). Moreover, we found that Sohlh2 overexpression increases the expression of LXRα, ABCA1 and ABCG1 in macrophages, while silencing of Sohlh2 inhibited the expression of LXRα, ABCA1 and ABCG1 (Fig. 4F–H). These results indicate that Sohlh2 plays a critical role in regulating cholesterol homeostasis, by activating LXRα/ABCA1, ABCG1 signaling pathway in macrophages.

### LXRα mediates the effects of Sohlh2 on M2 macrophage polarization and oncogenic properties of TNBC cells

**Fig. 5:**
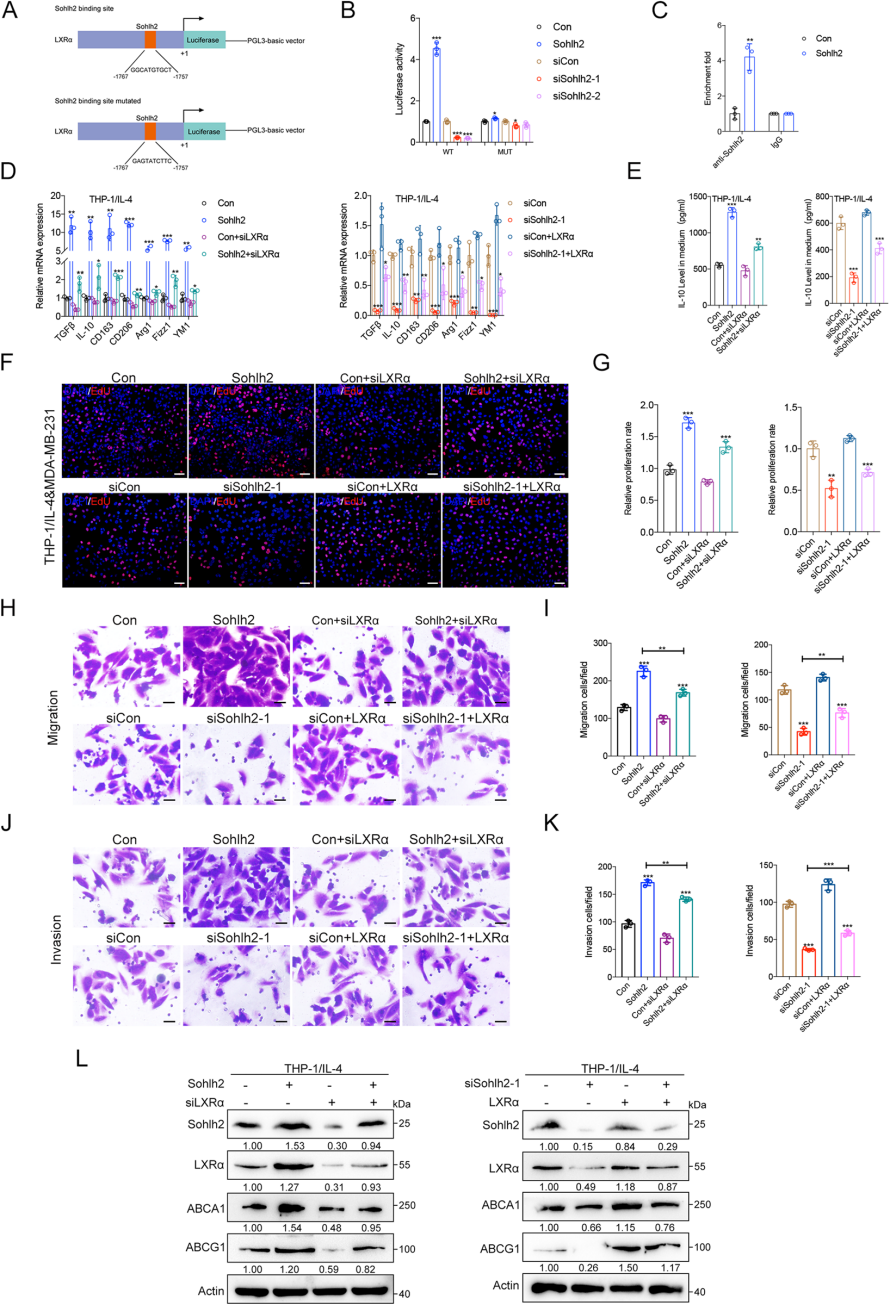
LXRα mediates the effects of Sohlh2 on M2 macrophage polarization and oncogenic properties of TNBC cells. **A** Schematic diagram of the luciferase reporter construct containing the Sohlh2 promoter and the mutant construct (LXRα-Wt and Mut). **B** LXRα promoter luciferase reporter activity in Sohlh2 overexpression and Sohlh2 siRNA THP-1 cells. **C** ChIP analysis of forced Sohlh2 expression THP-1 cells using anti–Sohlh2 antibody for LXRα promoter. **D**, **E** qPCR and Elisa analysis of the expression of M2 macrophage–related markers in Sohlh2-overexpressing THP-1 cells transfected with siLXRα plasmid. **F**, **G** The cells growth ability was measured by EdU assay. **H**–**K** Migration and invasion ability of the MDA-MB-231 cells co-cultured with Sohlh2 overexpression or knockdown THP-1 cells stably expressing siLXRα and LXRα by Transwell assays. **L** Western blot assay showed the expression of LXRα, ABCA1 and ABCG1 in Sohlh2 overexpression or knockdown M2 macrophages stably expressing siLXRα and LXRα. Scale bar indicates 50 μm. The values indicate the mean ± SD of three independent experiments. *P < 0.05, **P < 0.01, ***P < 0.001.

To further elucidate how Sohlh2 regulated LXRα transcription, ChIP and luciferase reporter assays were performed. According to the data of JASPAR (A database of transcription factor binding), LXRα promoter carries one predicted Sohlh2 DNA binding site, Sohlh2 overexpression increased the luciferase activity driven by the Luc-LXRα promoter construct, whereas mutating the binding site hardly altered the luciferase activity (Fig. 5A, B). ChIP assay was performed to confirm that Sohlh2 could directly bind to the promoter region of LXRα (Fig. 5C). To further investigate whether LXRα mediates the effects of Sohlh2 on macrophages, we generated THP-1 Sohlh2 overexpression or knockdown cells stably expressing siLXRα and LXRα, respectively. qPCR and Elisa assays showed that knockdown of LXRα significantly suppressed M2 macrophage polarization, while overexpression of LXRα promoted the expression of genes related to M2 macrophage, compared to the respective controls (Fig. 5D, E). As expected, LXRα knockdown in macrophages reversed the growth and invasion abilities enhanced by Sohlh2 (Fig. 5F, K). Similarly, LXRα restoration in macrophages rescued TNBC cell growth and invasion abilities reduced by Sohlh2 silencing (Fig. 5F, K). In addition, knockdown of LXRα attenuated the upregulation of the expression of ABCA1 and ABCG1 caused by Sohlh2 overexpression in THP-1 cells (Fig. 5L). Collectively, these data indicated that Sohlh2 promoted M2 macrophage polarization and TNBC progression via LXRα-dependent manner.

### TRIM21 mediates the ubiquitination and degradation of Sohlh2

**Fig. 6:**
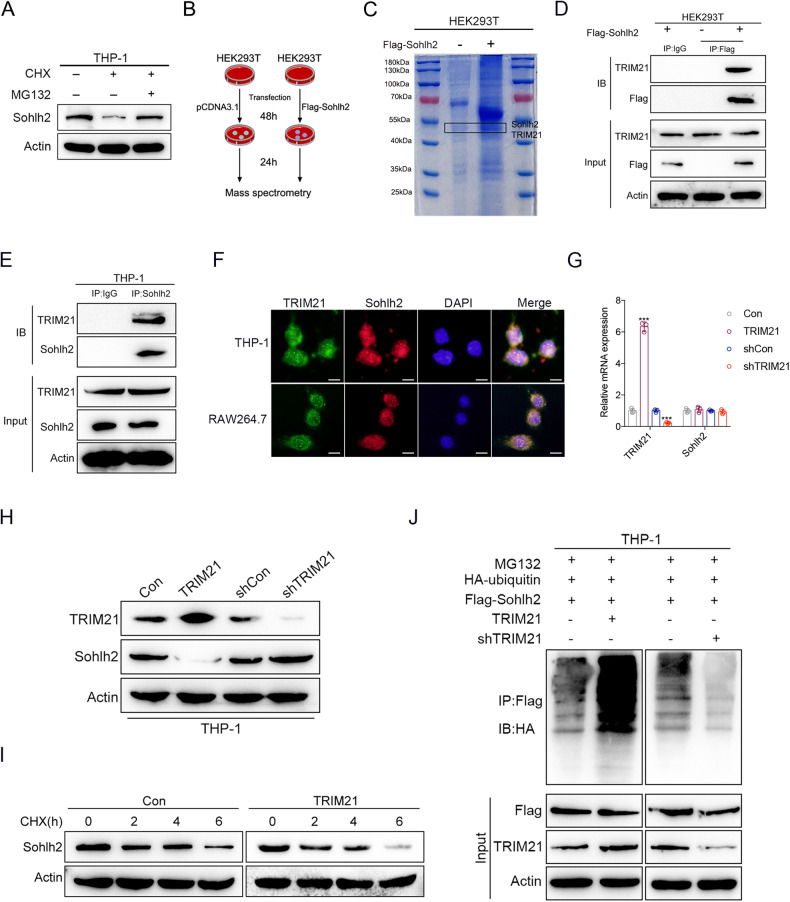
TRIM21 mediates the ubiquitination and degradation of Sohlh2. **A** THP-1 cells were treated with CHX (50 μg/ml) and MG132 (20 μM) either alone or in combination for 6 h, and Western blot assay showed that Sohlh2 degraded via the ubiquitinproteasome pathway. Mass spectrometry (**B**, **C**) and Co-IP assay (**D**, **E**) were performed to detect the interactive proteins with Sohlh2. **F** The co-localization of TRIM21 and Sohlh2 was determined by immunofluorescent analysis. qPCR assay (**G**) and Western blot assay (**H**) showed the expression of Sohlh2 in TRIM21 overexpressing THP-1 cells. **I** THP-1 cells were treated with CHX (50 μg/ml) for different times, and the effect of TRIM21 on the half-life of Sohlh2 was analyzed by Western blot. **J** THP-1 cells were transfected as indicated, and coimmunoprecipitation and Western blot analysis were performed to detect the role of TRIM21 on the ubiquitination of Sohlh2. Scale bar indicates 20 μm. The values indicate the mean ± SD of three independent experiments. *P < 0.05, **P < 0.01, ***P < 0.001.

The process of ubiquitination and degradation of the proteins is involved in protein stability. Immunoblots showed that proteasome degradation of Sohlh2 was blocked by the specific proteasome inhibitor (MG132) treatment (Fig. 6A). Then, we performed mass spectrometry and Co-IP assay to examine the partners of Sohlh2 protein. The results showed that Sohlh2 interacted with ubiquitin E3 ligase TRIM21(Fig. 6B–E). The interaction of Sohlh2 and TRIM21 was confirmed by immunofluorescent co-localization analysis (Fig. 6F). Thus, we examined the mRNA and protein stability of Sohlh2 in TRIM21 over-expressing cells and found that TRIM21 significantly reduced the protein levels of Sohlh2, but little affected its mRNA levels (Fig. 6G, H). Moreover, the degradation of Sohlh2 protein was significantly accelerated by TRIM21 in the presence of protein synthesis inhibitor (CHX) (Fig. 6I), suggesting that TRIM21 may mediate Sohlh2 protein ubiquitination and degradation. Ubiquitination assays showed that TRIM21 expression increased Sohlh2 ubiquitination levels in the presence of MG132, while knockdown TRIM21 attenuated Sohlh2 ubiquitination levels in THP-1 cells (Fig. 6J). These data indicate that TRIM21 promotes the ubiquitination and degradation of Sohlh2.

### TRIM21 suppresses M2 macrophage polarization and oncogenic properties of TNBC cells partly through a Sohlh2-dependent manner

**Fig. 7:**
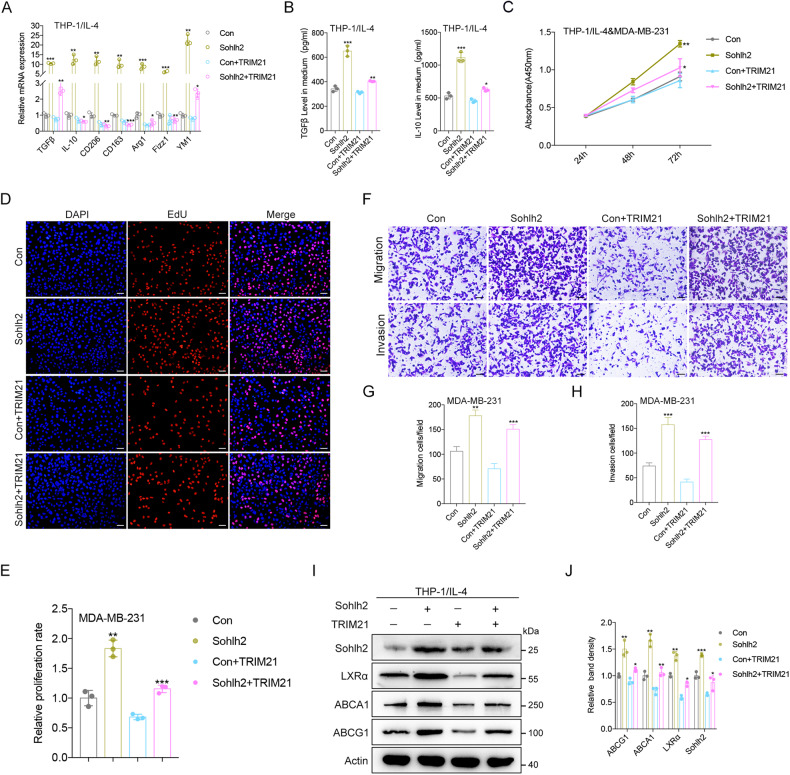
TRIM21 suppresses M2 macrophage polarization and oncogenic properties of TNBC cells partly through a Sohlh2-dependent manner. **A**, **B** qPCR and Elisa analysis of the expression of M2 macrophage–related markers in Sohlh2-overexpressing THP-1 cells with ectopic TRIM21. **C**–**E** The MDA-MB-231 cells co-cultured with Sohlh2 overexpression THP-1 cells stably expressing TRIM21. The growth ability was measured by EdU assay. **F**–**H** Migration and invasion ability of the MDA-MB-231 cells was detected by Transwell assays. **I**, **J** Western blot assay showed the expression of LXRα, ABCA1 and ABCG1 in Sohlh2 overexpression M2 macrophages stably expressing TRIM21. Scale bar indicates 50 μm. The values indicate the mean ± SD of three independent experiments. *P < 0.05, **P < 0.01, ***P < 0.001.

To investigate whether TRIM21 affect the biological function of Sohlh2 in M2 macrophage, qPCR and Elisa analysis were performed to illuminate that the expression of genes related to M2 macrophage was significantly decreased in TRIM21 and Sohlh2-overexpressing macrophages (Fig. 7A, B). Furthermore, TNBC cell proliferation and invasion properties were significantly restrained when cocultured with TRIM21 and Sohlh2-overexpressing macrophages (Fig. 7C–H). In addition, the protein levels of LXRα, ABCA1 and ABCG1 were repressed in TRIM21 and Sohlh2-overexpressing macrophages (Fig. 7I, J). Collectively, these results indicate that TRIM21 suppresses M2 macrophage polarization and growth, migration, and invasion of TNBC cells partly through a Sohlh2-mediated way.

### Sohlh2 is correlated with the expression of CD163 and LXRα in TNBC-associated macrophages

**Fig. 8:**
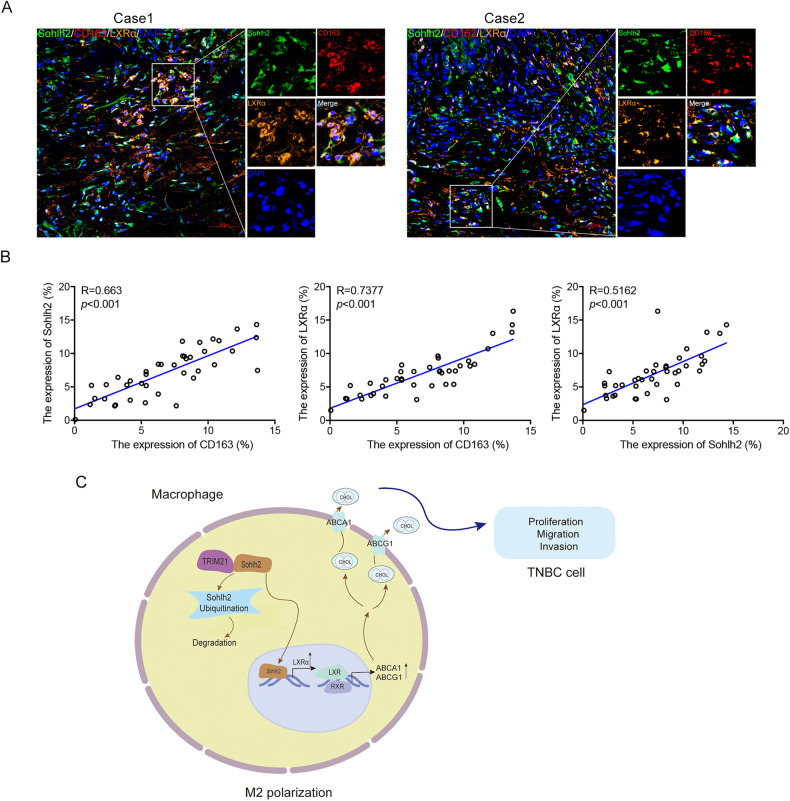
Sohlh2 is correlated with the expression of CD163 and LXRα in TNBC-associated macrophages. **A**, **B** The immunofluorescence assay was performed to detect the expression of Sohlh2, CD163 and LXRα in TNBC tissues. **C** A schematic model showed the role of the TRIM21/Sohlh2/LXRα signaling in the regulation of M2 macrophage polarization and progression of TNBC. Scale bar indicates 50 μm.

To evaluate the role of Sohlh2 in TAMs of TNBC, the relevance of Sohlh2, CD163 and LXRα expression was further investigated in human TNBC tissues. Confocal microscopy demonstrated that CD163, Sohlh2 and LXRα protein expression were assessed in 40 cases of TNBC tissues characterized for Sohlh2 expression and subdivided into Sohlh2-positive TAMs and Sohlh2-negative TAMs. The correlation of Sohlh2 and clinical characteristics was shown in Supplementary Table S4. A positive correlation between CD163, Sohlh2 and LXRα was observed in TAMs (Fig. 8A, B). A schematic model showed the role of the TRIM21/Sohlh2/LXRα signaling in the regulation of M2 macrophage polarization and progression of TNBC (Fig. 8C). Collectively, these results suggest that the regulation of macrophage M2 polarization and TNBC progression is mediated by TRIM21/Sohlh2/LXRα signaling pathway.

## Discussion

Tumor microenvironment (TME) has been recognized to play a critical role in tumor development and metastasis. Within the tumor stroma, a significant entity known as TAMs has captured increased attention in breast cancer treatment [26, 27]. TAMs hold the distinction of being the most abundant immune cells within the TME, serving as key constituents of inflammatory circuits that propel the progression and metastasis of tumors [28, 29]. Recent studies have focused on better understanding and possibly targeting the microenvironment in which tumors develop and metastasis [30, 31]. Notably, lung metastasis stands as a formidable culprit behind the mortality experienced by patients with TNBC [32, 33].

In the present study, we demonstrated that Sohlh2 was highly expressed in M2 macrophages. Importantly, we identified Sohlh2 promoted the polarization of macrophages towards the M2 phenotype. In an orthotopic xenograft model of TNBC, we revealed a metastasis-promoting function for macrophage-specific Sohlh2 KI mice in vivo, which was mediated in part by convening Sohlh2-dependent M2 polarization of macrophages in the primary tumor. Our findings provided evidence that Sohlh2 in TAMs may serve as a promising therapeutic target for treatment lung metastasis in TNBC.

It has been documented that cholesterol metabolism exerts influence on the induction of inflammatory responses in macrophages [11, 34]. In IL-4-stimulated macrophage, an intriguing protein known as FAMIN facilitates a connection between de novo lipogenesis (DNL) and fatty acid oxidation (FAO), creating an apparent “substrate cycle” that potentially enhances oxidative metabolic flux to support bioenergetics [11]. The activation of the cholesterol efflux pathway by LXRs is pivotal in safeguarding cells against lipid overload [16, 35, 36]. LXRs serve as cholesterol sensors in macrophages, holding significant importance in maintaining the scavenging function of these cells. Additionally, LXRs regulate macrophage inflammatory responses, repressing the expression of genes encoding inflammatory cytokines, such as TNFα and IL-1β [37, 38]. LXRα and LXRβ, members of the nuclear receptor family, play a critical role in regulating cholesterol homeostasis [15, 16]. Comparatively, our findings indicate that Sohlh2 exerts a more substantial regulatory influence on LXRα as opposed to LXRβ (Fig. S2C). Interestingly, we also found deficiency in LXRα could reverse the promotion of Sohlh2 on TNBC cell migration and invasion when co-cultured with macrophages. These results suggest a mechanism in which Sohlh2 functions as a factor promoting M2 polarization, increasing TNBC lung metastasis through the upregulation of LXRα in macrophages. LXRα plays a critical role in regulating the reverse cholesterol transport process through its control over the expression of key cholesterol transporters and apolipoproteins, including ABCA1, ABCG1, apoE, and apoC [11, 19, 39]. Sohlh2 concurrently orchestrated the expression of LXRα, ABCA1, and ABCG1. Furthermore, this orchestration disrupted the lipid homeostasis on the macrophage membrane. Moreover, ChIP and dual luciferase assays provided compelling evidence of direct binding between Sohlh2 and the promoter region of LXRα, thereby enhancing its transcriptional activity. Notably, we identified a specific DNA binding site for Sohlh2, represented by an E box sequence, within the LXRα promoter region. Introduction of single-site mutations in this sequence resulted in a substantial reduction in promoter activity compared to the wild-type control. These findings establish LXRα as a direct target of Sohlh2, mediating the effects of Sohlh2 on macrophage polarization and lung metastasis.

The process of ubiquitination-deubiquitination of proteins is a key contributor in regulating protein localization, stability, activity, and interaction with other proteins [40, 41]. Extensive evidence supports the notion that E3 ligases serve as oncogenes or tumor suppressors, depending on the substrate function [42–45]. While our study did not reveal a significant upregulation of Sohlh2 at the mRNA level upon TRIM21 overexpression, it is plausible that TRIM21 regulates Sohlh2 through post-translational modifications. Intriguingly, TNBC cells overexpressing TRIM21 treated with CHX, were observed a significant acceleration in the degradation of Sohlh2 protein mediated by TRIM21. It was identified that TRIM21 may attenuate the stability of Sohlh2 protein. We found that there was an interaction of Sohlh2 and TRIM21, and overexpression of TRIM21 significantly increased the ubiquitination of Sohlh2. Moreover, TRIM21 overexpression in macrophages suppressed macrophage polarization towards an M2-like phenotype, TNBC cell proliferation, migration and invasion induced by forced expression of Sohlh2. These findings suggest that TRIM21 functions as a tumor suppressor by attenuating Sohlh2 protein levels, thereby inhibiting M2 macrophage polarization.

In conclusion, our study identified Sohlh2 as a specific regulator of M2 macrophage polarization, exerting its effects through the transcriptional regulation of LXRα. Sohlh2/LXRα signaling pathway enhanced lipid efflux from macrophages and disrupted the lipid balance in the cellular membrane, which was involved in macrophage M2 polarization. Sohlh2 overexpression effectively accelerated macrophage-mediated TNBC cell metastasis both in vitro and in vivo. Furthermore, TRIM21 not only elevated the ubiquitination and degradation of Sohlh2, but also altered the impact of Sohlh2 on macrophage polarization. Our work unveils the role of TRIM21/Sohlh2/LXRα signaling pathway in regulating macrophage M2 polarization and TNBC progression, suggesting its potential relevance to clinical conditions associated with M2 macrophage polarization.

### Supplementary information

Supplementary materials

checklist

Original Data File

## Data Availability

The datasets generated or analyzed during the current study are included within the article and available from the corresponding authors on reasonable request.
