# Supplementary material for: TRIM21-mediated Sohlh2 ubiquitination suppresses M2 macrophage polarization and progression of triple-negative breast cancer
Source: Cell Death Dis. 2023 Dec 20;14(12):850. doi: 10.1038/s41419-023-06383-x (PMC10733312; doi:10.1038/s41419-023-06383-x)
Supplement: Supplementary file 1 — Supplementary materials [file 41419_2023_6383_MOESM1_ESM.docx]

**Supplementary materials**


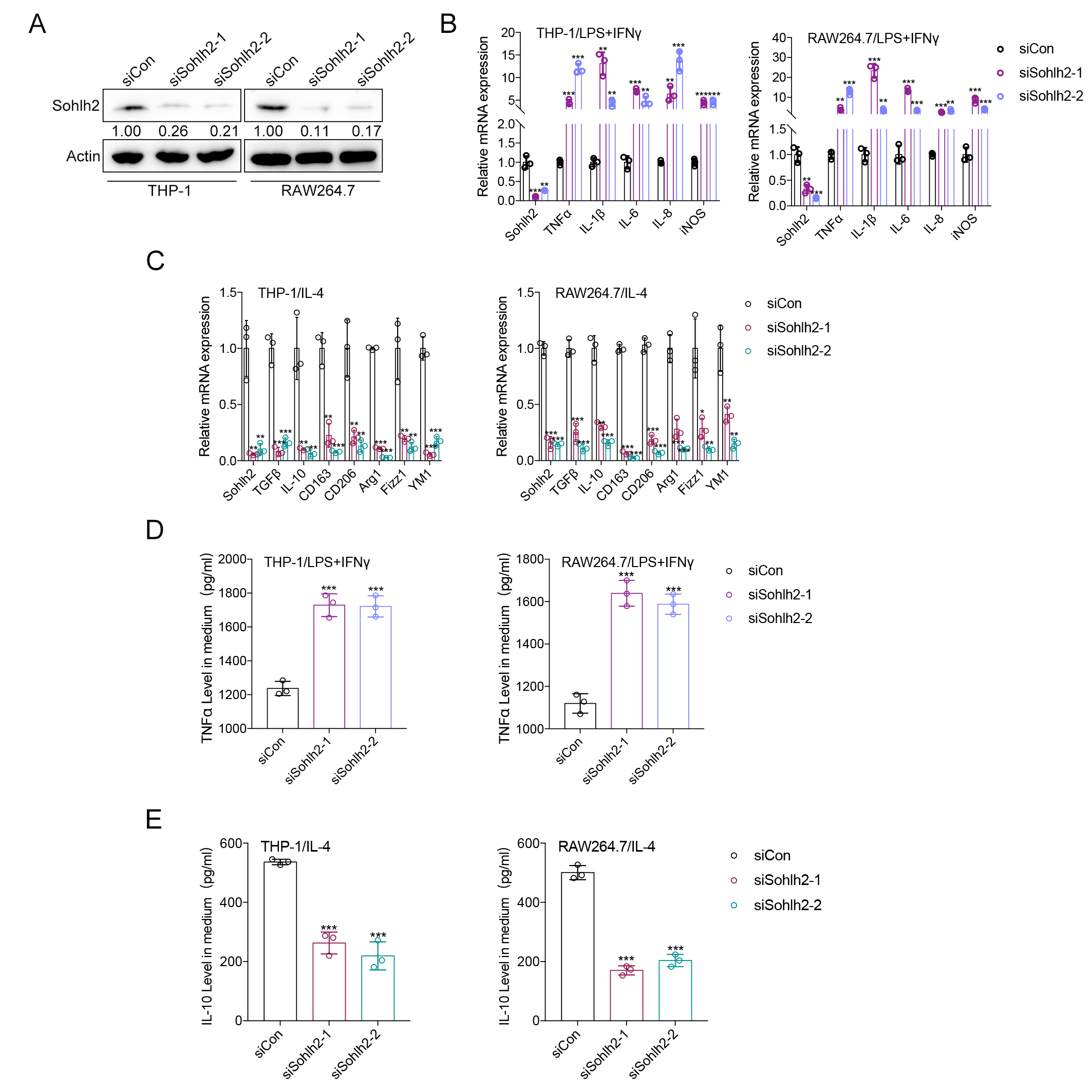


**Supplementary Figure 1.** Knockdown Sohlh2 repressed M2 macrophage polarization. **A** Western blot assay showed the efficiency of Sohlh2 knockdown in macrophage. **B, C** The mRNA expression levels of M1 or M2 macrophage-related genes were examined by qPCR. **D, E** The secretion of TNFα and IL-10 was measured by Elisa analysis. The values indicate the mean ± SD of three independent experiments. **P* < 0.05, ***P* < 0.01,****P* < 0.001.


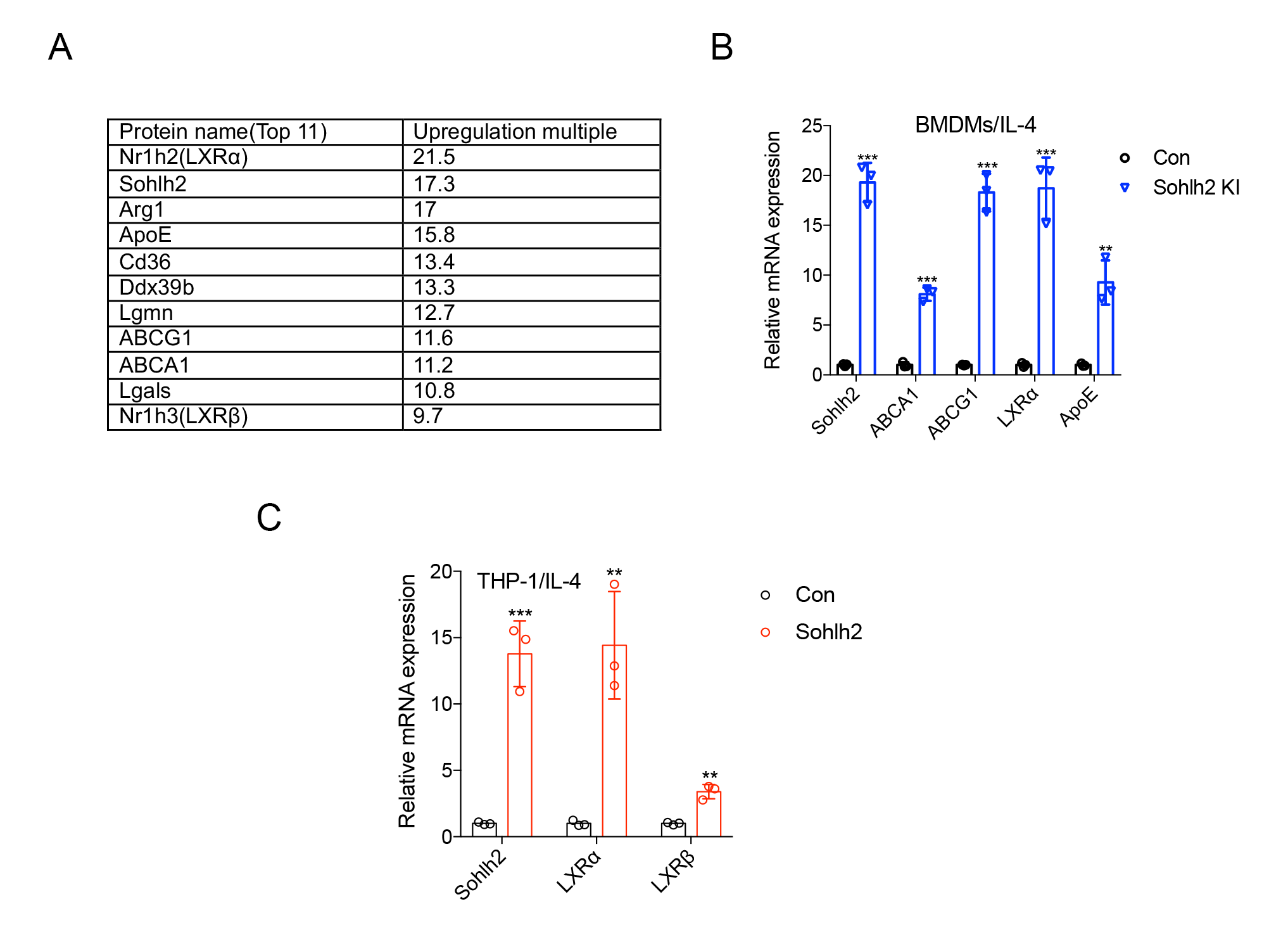


**Supplementary Figure 2.** LXRα was the highest upregulated gene. **A** The top 11 upregulated genes identified in Sohlh2 KI BMDMs by mass spectrometry. **B** qPCR analysis of LXRα, ABCA1 and ABCG1 in BMDMs. **C** qPCR analysis of LXRα and LXRβ in THP-1 cells. The values indicate the mean ± SD of three independent experiments. **P* < 0.05, ***P* < 0.01,****P* < 0.001.

**Supplementary Table 1:** The target sequences selected are shown as follows:

| siRNA | target sequences (5’-3’) |
| --- | --- |
| siSohlh2-1 | GCUCCAAUUCCUGACUAAUAC |
| siSohlh2-2 | UCUCCUGCCGUUAUGGCCCAGAUUA |
| si-m-Sohlh2-1 | CGTTGTCGAGTCTAGACAA |
| si-m-Sohlh2-2 | GAAGGTGACTTCCACTCTA |
| siLXRα | AGUUCUCCAGGGCCAUGAAUG |
| shTRIM21 | TGGCATGGTCTCCTTCTACAA |

**Supplementary Table 2:** Primer sequence for qPCR

| Gene | Sequence |
| --- | --- |
| GAPDH-F | GAAAGCCTGCCGGTGACTAA |
| GAPDH-R | AGGAAAAGCATCACCCGGAG |
| Sohlh2-F | CGGCCATGGCTTCCTCAAT |
| Sohlh2-R | AGTATCAGCCAGGTAGCCCA |
| LXRα-F | TCTGGACAGGAAACTGCACC |
| LXRα-R | AAGGCACTGTCCAAATCCCC |
| ABCA1-F | CCACCAATCTGCCTGTGCTA |
| ABCA1-R | AAGAGGTTCACGCTGGTGAG |
| ABCG1-F | TGTCTGATGGCCGCTTTCTC |
| ABCG1-R | CTGGACACCACCTCATCCAC |
| LXRβ-F | CAGAGCGCAAGCGAAAGAAG |
| LXRβ-R | GCTGAGCACGTTGTAGTGGA |
| m-GAPDH-F | AAGAGGGATGCTGCCCTTAC |
| m-GAPDH-R | GTTCACACCGACCTTCACCA |
| m-Sohlh2-F | AGCTGAGACGAGAACGCATC |
| m-Sohlh2-R | GGCCTCGATAACTGATGCCA |
| m-LXRα-F | CAAAGAGCCTCCAGGGTGAG |
| m-LXRα-R | TCCCTCTACCAAAACTGTCGC |
| m-ABCA1-F | TAGCAGCACCGTGTCTTGTC |
| m-ABCA1-R | GCGTGTCACTTTCATGGTCG |
| m-ABCG1-F | GGTCCTGACACATCTGCGAA |
| m-ABCG1-R | CAGGACCTTCTTGGCTTCGT |
| LXRα promoter-F | ACTTGTAGTCCCAGCTACTTGGGAT |
| LXRα promoter-R | CCCGGCCCAAAATATATACATATAT |

**Supplementary Table 3:**The list of antibodies used for Western blot analysis.

| Parameter | Producer | Dilution |
| --- | --- | --- |
| Sohlh2 | Novus- | 1:200(IF); 1:1000(WB) |
| LXRα | Affinity-DF6864 | 1:100(IF); 1:1000(WB) |
| ABCA1 | Affinity-DF8233 | 1:500(WB) |
| ABCG1 | Affinity-DF7693 | 1:1000(WB) |
| CD163 | Abcam-ab182422 | 1:200(IF) |
| E-cadherin | CST-#14472 | 1:1000(WB) |
| N-cadherin | CST-#13116 | 1:1000(WB) |
| Vimentin | CST-# 5741 | 1:1000(WB) |
| Occludin | Affinity-DF7504 | 1:1000(WB) |
| iNOS | Abcam- ab178945 | 1:200(IF) |
| Actin | Affinity-AF7018 | 1:1000(WB) |
| TRIM21 | CST#92043 | 1:200(IF); 1:1000(WB) |
| HA-Tag | Sigma-h3663 | 0.5 μg/mL |

**Supplementary Table 4: The correlation of Sohlh2 expression in TNBC TAMs and clinical characteristics**

**
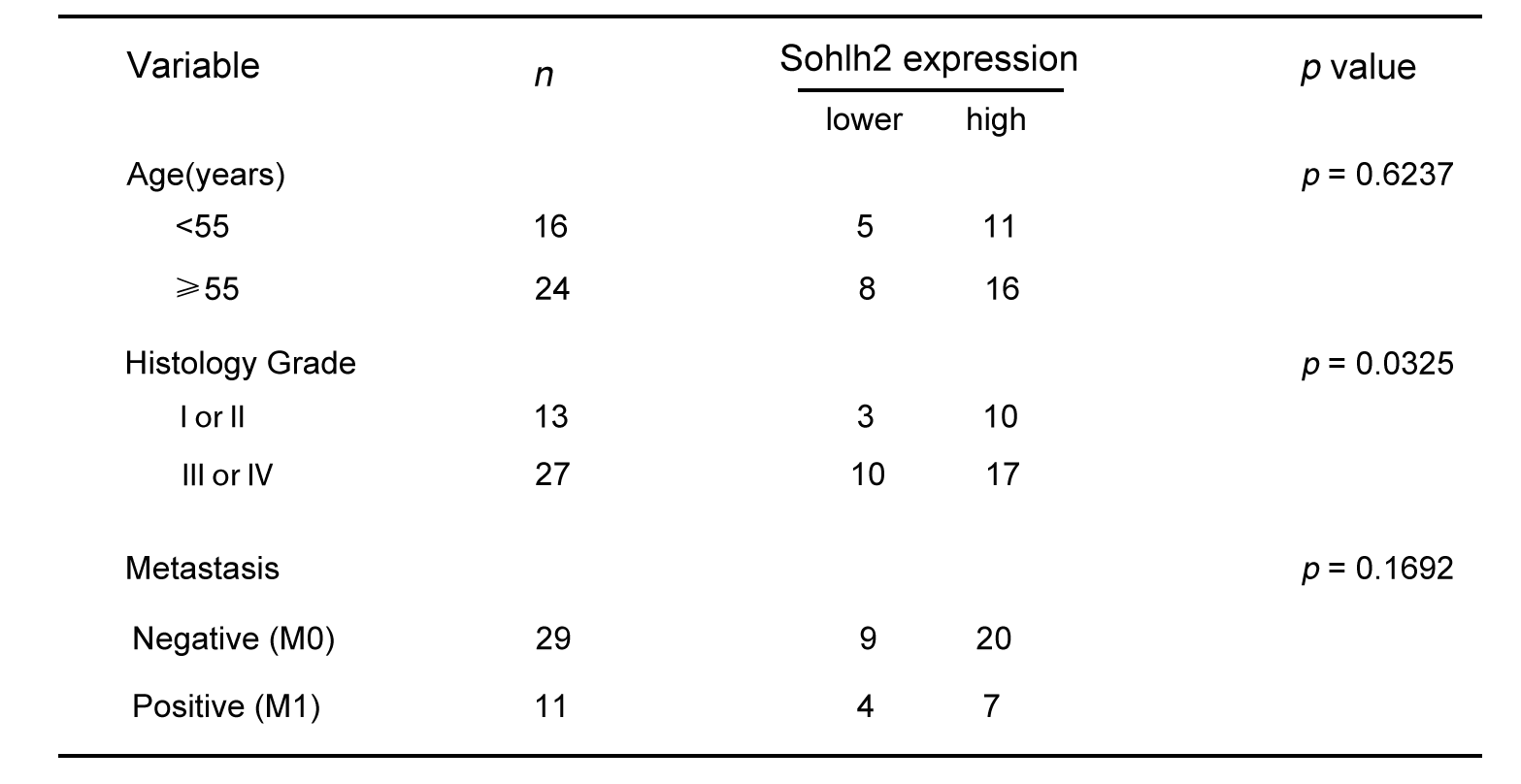
**
