# Supplementary material for: TRIM21-mediated Sohlh2 ubiquitination suppresses M2 macrophage polarization and progression of triple-negative breast cancer
Source: Cell Death Dis. 2023 Dec 20;14(12):850. doi: 10.1038/s41419-023-06383-x (PMC10733312; doi:10.1038/s41419-023-06383-x)

Original western blot of Figure 1


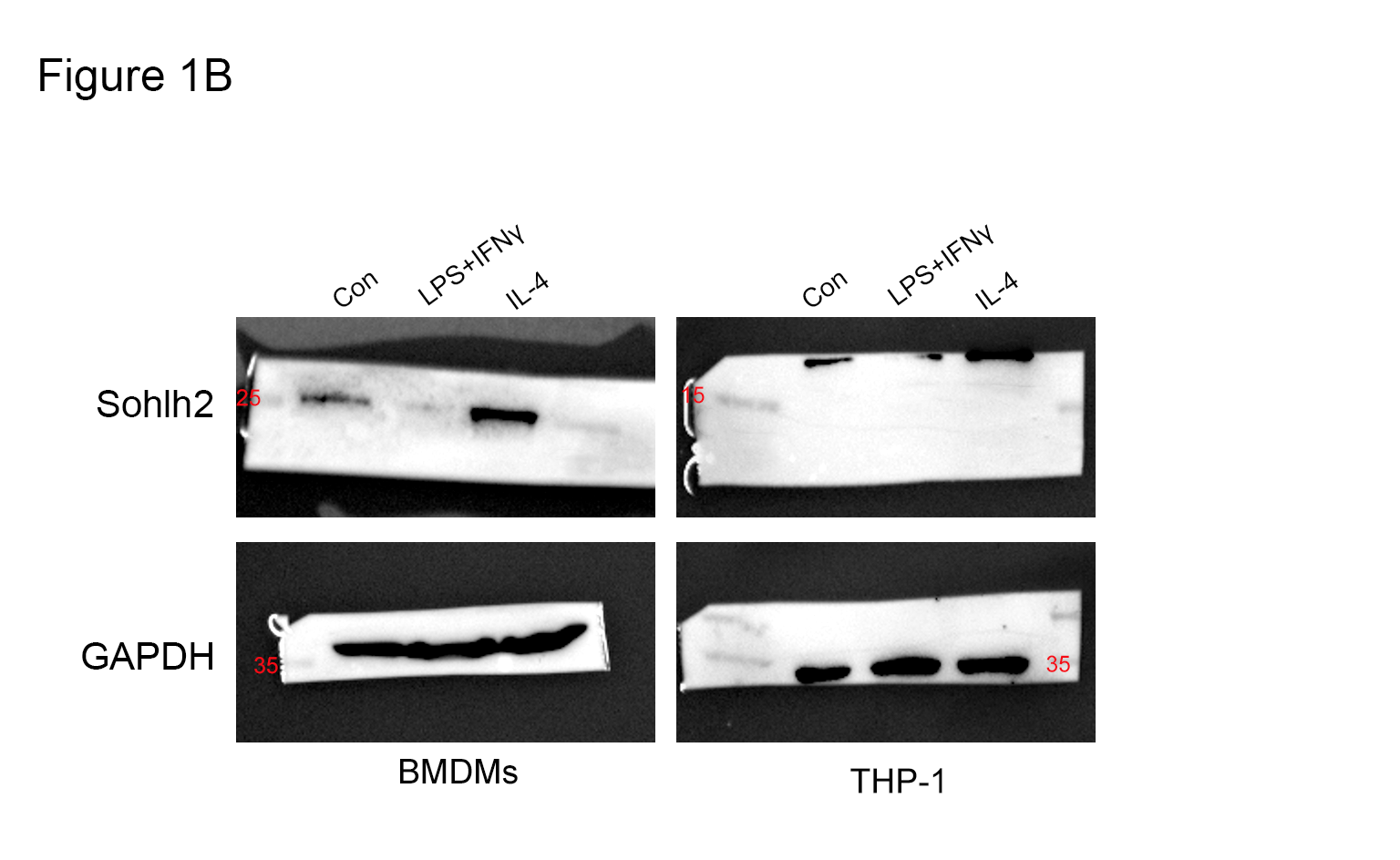


Original western blot of Figure 2


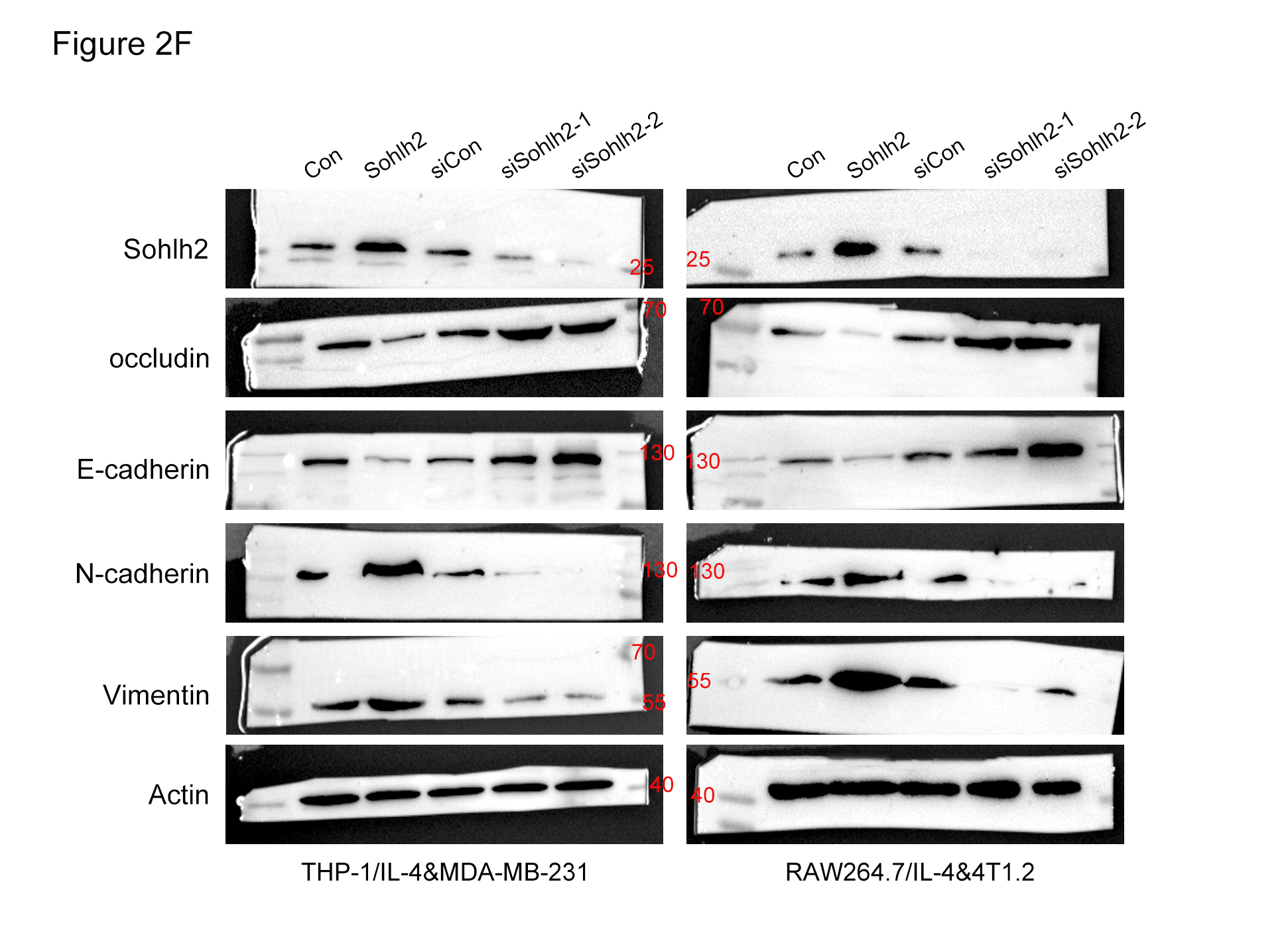


Original western blot of Figure 4


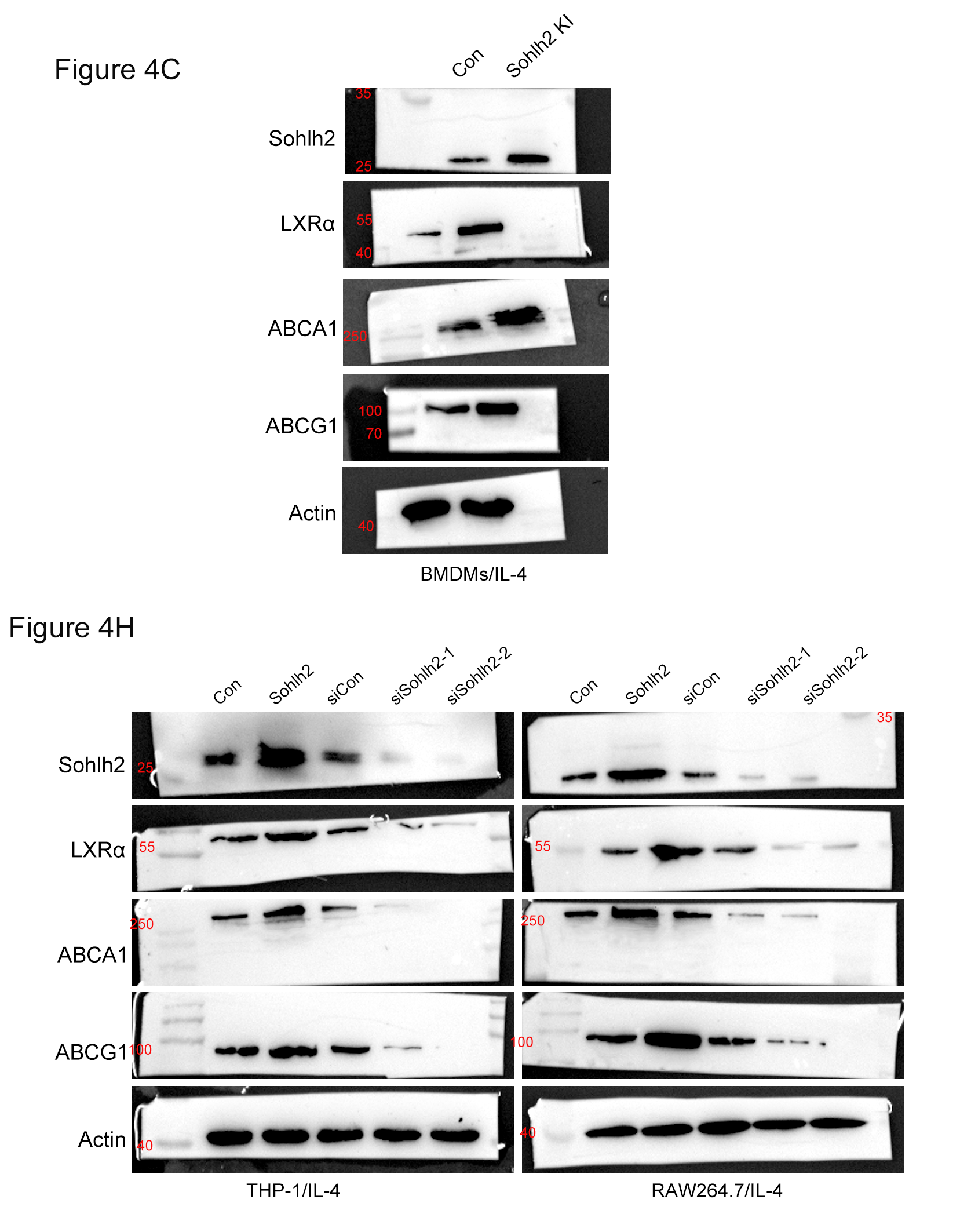


Original western blot of Figure 5


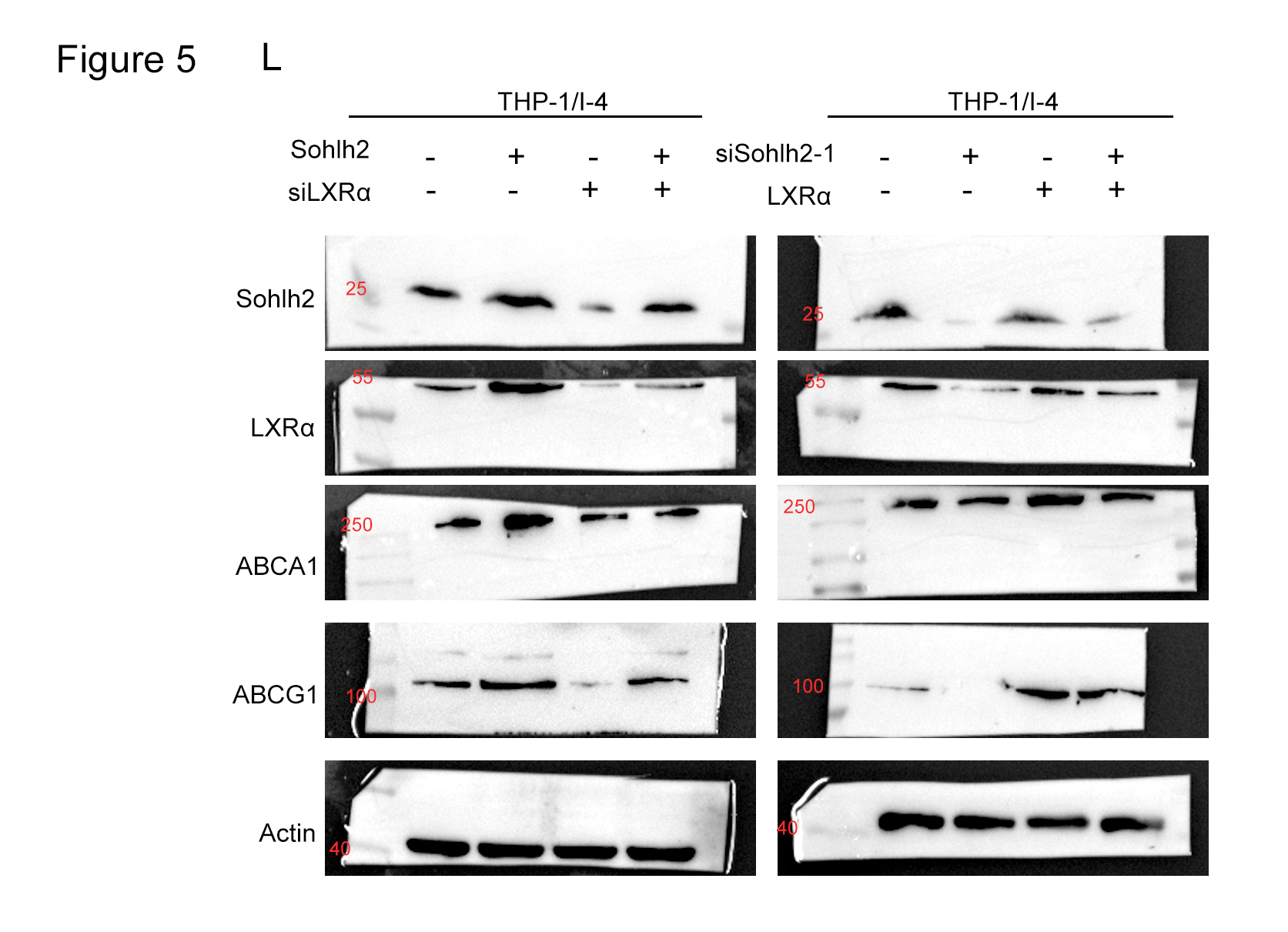


Original western blot of Figure 6


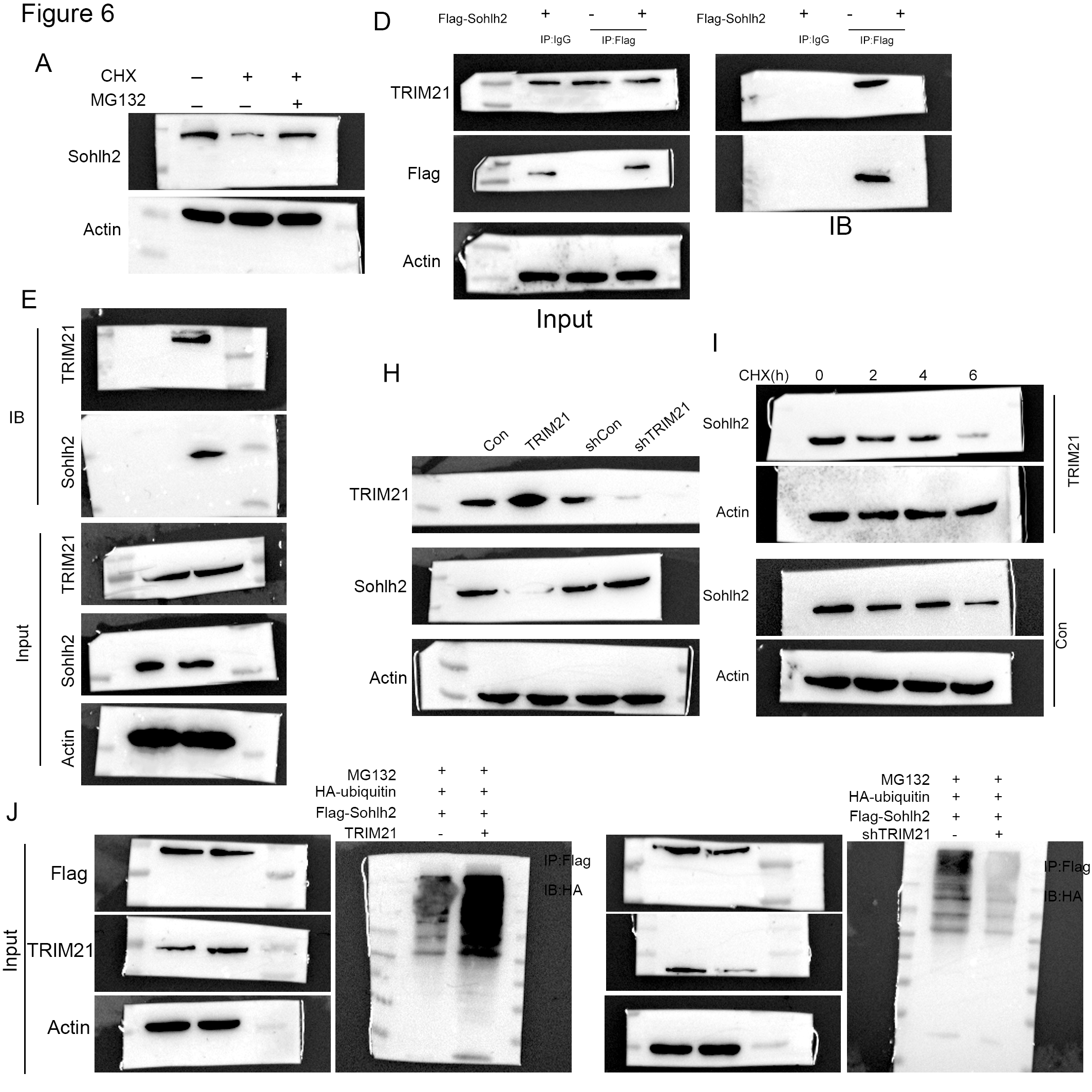


Original western blot of Figure 7


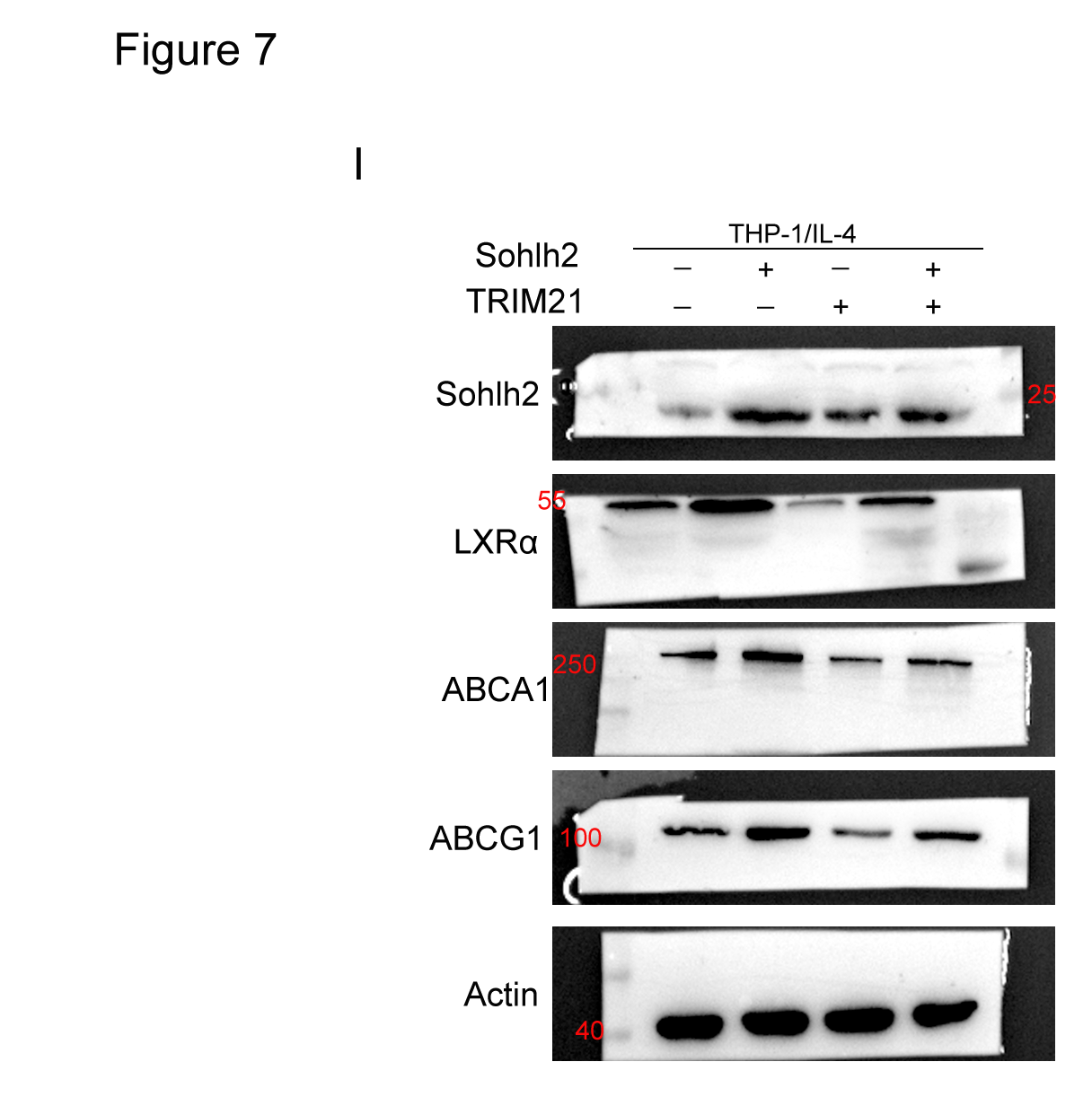

Supplement: Supplementary file 3 — Original Data File [file 41419_2023_6383_MOESM3_ESM.docx]
